# Colpocleisis as an obliterative surgery for pelvic organ prolapse: is it still a viable option in the twenty-first century? Narrative review

**DOI:** 10.1007/s00192-021-04907-7

**Published:** 2021-08-18

**Authors:** Magdalena Emilia Grzybowska, Konrad Futyma, Aida Kusiak, Dariusz Grzegorz Wydra

**Affiliations:** 1 Department of Gynecology, Gynecologic Oncology and Gynecologic Endocrinology, Medical University of Gdańsk, Smoluchowskiego 17, 80-214 Gdańsk, Poland; 2 2nd Department of Gynecology, Medical University of Lublin, Lublin, Poland; 3 Department of Periodontology and Oral Mucosa Diseases, Medical University of Gdansk, Orzeszkowej 18, 80-204 Gdansk, Poland

**Keywords:** Colpocleisis, Complications, Pelvic organ prolapse, Regret, Satisfaction, Success

## Abstract

**Introduction and hypothesis:**

The aims were to review the literature from the last two decades and analyze treatment efficacy and findings of the studies on colpocleisis.

**Methods:**

A systematic search was conducted within the MEDLINE/PubMed and ClinicalTrials.gov databases, using the following keywords: pelvic organ prolapse (POP), colpocleisis, obliterative, and LeFort. All English full-text prospective and retrospective observational and interventional studies were included. Anatomical and subjective success, surgical techniques, concomitant procedures, complication rates, anesthesia methods, and decision regret were analyzed.

**Results:**

A total of 237 papers were identified and 49 met the inclusion criteria. Mean patient age was 69.0 ± 8.0 to 84 ± 3.1. Over 90.2% of patients undergoing colpocleisis were diagnosed with POP stage ≥ 3. The follow-up ranged from 30 days to a median of 5 years. Anatomical success, defined as POP-Q stage ≤ 1 and no prolapse beyond the hymen, was achieved in 62.5 to 100% and 87.5 to 100% of all patients respectively. Subjective success ranged from 88% to 100%. Regret over the loss of coital ability ranged from 0% in many studies to 12.9%, general decision regret from 0% to 13.8%. After concomitant midurethral sling surgery, 86.8% to 94% of all patients were continent, with a 0–14% sling revision rate due to urinary retention. Urinary tract infection was the most common postoperative complication (4.3 to 9% confirmed with urine culture, 34.7% based on symptom definition). Bowel (0 to 2.7%) and urinary tract (0 to 9.1%) injuries were the consequences of concomitant procedures. The mortality rates were up to 1.3%.

**Conclusions:**

Colpocleisis is a heterogeneous procedure, characterized by high subjective and objective success, low coital ability regret, and a low risk of complications.

**Supplementary Information:**

The online version contains supplementary material available at 10.1007/s00192-021-04907-7

## Introduction

Surgical intervention for pelvic organ prolapse (POP) repair is associated with a choice between reconstructive and obliterative surgery. Native tissue repairs and mesh-augmented procedures are used in reconstructive surgery, whereas obliterative surgery may be considered in some patients, especially those with numerous concomitant diseases and no desire for sexual activity in the future. As colpocleisis renders vaginal intercourse impossible, it is predominantly recommended for the elderly. Colpocleisis can be performed with the use of local anesthesia [1, 2].

In the nineteenth century, surgical obliteration of the vagina was introduced as a treatment for POP. The procedure was first performed by Neugebauer of Warsaw (in 1867) and later performed and published by LeFort of Paris (in 1877). Originally, it did not include a concomitant hysterectomy [3, 4]. During surgery, the vaginal epithelium is dissected off the underlying fibromuscular layers anteriorly and posteriorly, with or without leaving epithelial strips on the sides to create tunnels of drainage if the uterus is preserved. The anterior and the posterior denuded walls are sewn together, either with purse-string or horizontal rows of interrupted sutures, a few centimeters above the hymen [4]. After the vagina has been inverted, the superior and the inferior margins of the vagina are sutured horizontally. In order to reduce the genital hiatus, perineal repair and/or levator plication is often performed concomitantly, although it is not an inherent part of the procedure [4]. Some modifications of the technique include using a synthetic or a biological graft between the anterior and the posterior vaginal walls [2]. Occult stress urinary incontinence (SUI) is reported in almost 70% of all women with advanced prolapse [5]. Therefore, many researchers assess the results of the anti-incontinence procedure performed concomitantly with colpocleisis [6]. One study demonstrated that the denudation of the vagina with razor-type dermatomes allowed the preservation of a thicker fibromuscular layer of the pubocervical and rectovaginal fascia and reduction of operative time [7]. Despite having a considerable history, the search for improvements to the procedure of colpocleisis continues, aiming to ensure high efficacy and low risk of complications and side effects.

Opponents of the procedure claim that, owing to irreversible loss of vaginal coital function, colpocleisis is associated with significant post factum regret. However, reports of low regret rates and a positive impact on the pelvic symptoms have been published [8]. Another contentious aspect of colpocleisis, if the uterus is preserved, is the loss of the possibility of performing postoperative diagnostic tests for cervical or endometrial malignancy. Even though the risk of endometrial cancer seems too low to justify concomitant hysterectomy, some experts recommend evaluating the uterine cavity in asymptomatic patients before colpocleisis, with either ultrasound or sampling [3, 6]. However, in low-risk women, no endometrial evaluation before LeFort colpocleisis demonstrates superior cost utility [9].

The objective of this study was to review the literature on colpocleisis from the last two decades and to analyze treatment efficacy and the findings of those studies that may affect the decisions about POP management.

## Materials and methods

### Search strategy and eligibility

The search was performed using the MEDLINE/PubMed and ClinicalTrials.gov databases. We reviewed the literature from 2000 to 2020 on obliterative vaginal surgery performed for POP, and analyzed studies that investigated the risks and benefits as well as patient-reported outcomes of those procedures. The search terms were as follows: “pelvic organ prolapse,” “colpocleisis,” “obliterative,” and “LeFort.”

**Fig. 1:**
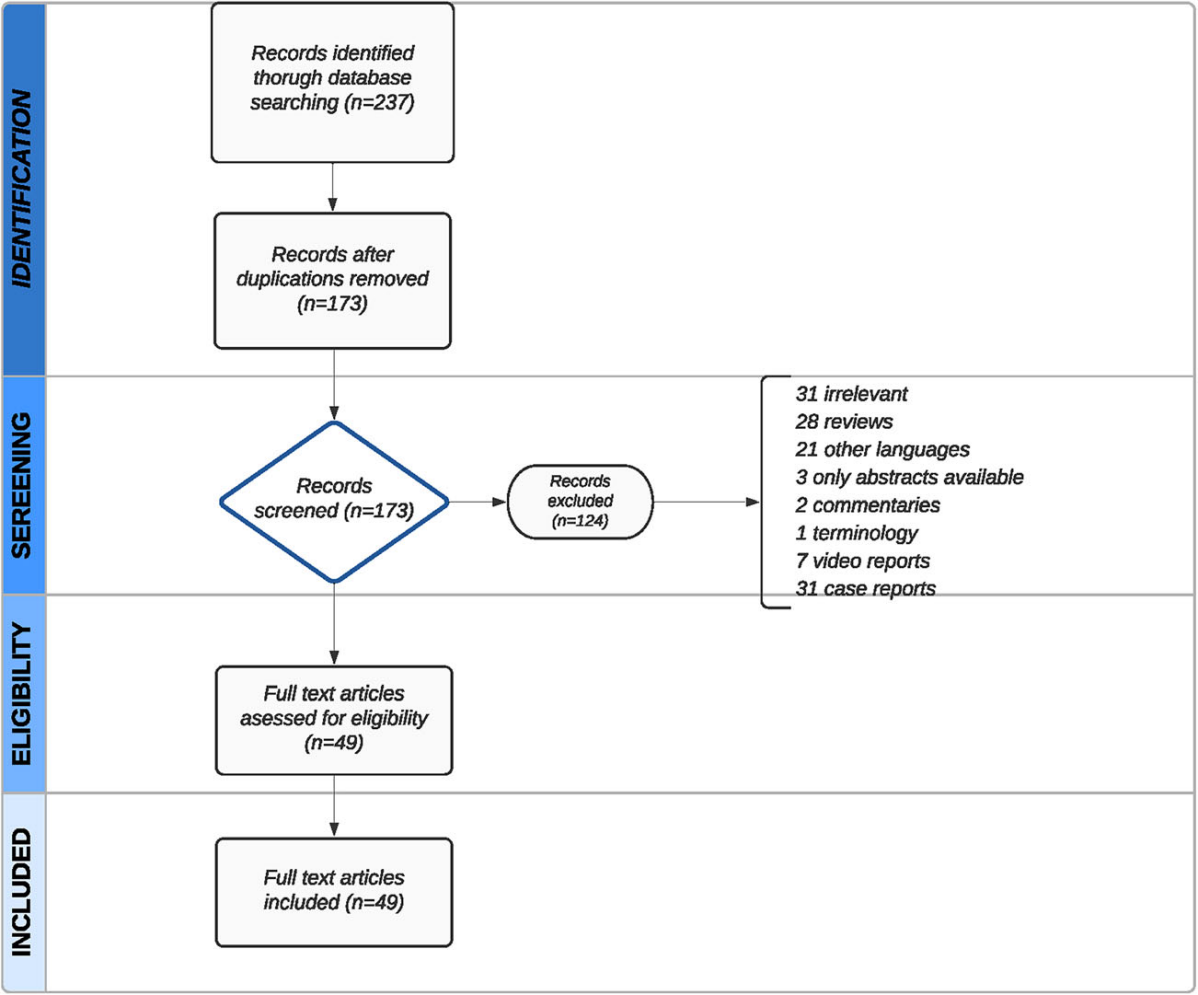
Preferred Reporting Items for Systematic Reviews and Meta-​Analyses flowchart of selection steps of articles

The articles were selected for further analysis by careful screening of the titles, abstracts, and full texts. Case reports, editorials, texts written in languages other than English, abstracts from international congresses or review articles were excluded from further analysis (Fig. 1). We included all full-text articles in English. The study type, research sample, and follow-up length were not restrictions. The last search was performed on 15 June 2020. The authors (MEG and KF) conducted the literature search independently, and any disagreement in an article’s inclusion was resolved after direct discussion between the authors.

The participants were women who underwent a colpocleisis procedure. The interventions were obliterative surgery/colpocleisis. Reconstructive surgery, dermatome dissection, and colpocleisis were compared with concomitant VH or with midurethral sling (MUS); however, most of the studies used only an intervention group. The primary outcome was the objective anatomical and patient-reported subjective success. The results were summarized in tables by category.

### Data extraction and outcomes

The following data were extracted and analyzed: patient characteristics (age, stage of prolapse), surgery modalities (types of colpocleisis, concomitant hysterectomy, and anti-incontinence procedures – MUS), type of anesthesia, peri- and postoperative complications, time of follow-up, recurrence and reoperation rates. The anatomical and patient-reported success were analyzed. Special attention was paid to regret rates (general and coital function), body image, and goal accomplishment assessment; papers on obliterative surgery in relation to reconstructive surgery were also assessed. Postoperative goal accomplishment was defined on a scale from strongly disagree to strongly agree.

Continuous variables were expressed as mean ± standard deviations, and categorical variables as percentages of the total group. The *p* value of < 0.05 was considered to be statistically significant. This review study did not require the approval of the Local Ethics Committee.

### Data items variability

In terms of terminology, obliterative prolapse repairs can be categorized as follows: colpocleisis with hysterectomy, colpocleisis without hysterectomy (LeFort colpocleisis), and colpocleisis of the vaginal vault [4]. Synonyms that are not recommended, but used very often, include partial or total colpocleisis, vaginal obliteration, vaginectomy, and colpectomy [4].

The follow-up in this group of patients presents a considerable challenge, typical of colpocleisis. It is associated with the age of the patients, who often do not live long enough to have any prolonged follow-up or whose severe cognitive impairment prevents them from submitting feedback. Therefore, as far as follow-up is concerned, survey studies often analyze only a part of the initial study sample. The definitions of success need to be divided into “anatomical success,” i.e., objective assessment during an office visit, and patient “self-reported subjective success.” In most studies, anatomical success is presented as POP-Q stage ≤1, whereas POP-Q stage 2 is the cut-off for recurrence. In other studies, success is described as no prolapse beyond the hymen or no recurrence (Table 1). Subjective success is typically evaluated with the use of questionnaires such as Patient Global Impression of Improvement (PGI-I), Pelvic Floor Distress Inventory (PFDI), Pelvic Floor Impact Questionnaire (PFIQ), and general subjective satisfaction question. More in-depth analyses use body image assessment and recommendation or regret questions. The Decision Regret Scale (DRS) has been applied in some studies.

**Table 1:** Studies presenting success criteria and clinical outcomes, types of colpocleisis, and concomitant procedures

References	*N*	Types of colpocleisis, *n* (%)		Concomitant hysterectomy, incontinence procedures, *n* (%)	Age, mean ± SD, (range)	Follow-up period(months)	Methods of outcome analysis, criteria for O and S success	Success rate
Kato et al. [10]	20 34	Total colpocleisis	Standard Dermatome	– –	75.2±5.9 76.0±6.4	12	O: POP-Q ≤I	O: 47/54(87.0%)
Villot et al. 2020 [10]	37	Hysterocolpectomy/vaginal vault repair and colpocleisis		11(29.7) MUS	81.2±6.0	Office 17 Survey 44	S: PGI-I much better/better PFDI-20	S: 26/29(89.7%) PFDI-20 significantly improved
Wadsworth et al. 2020 [11]	10	LeFort colpocleisis			(72–87)		Semi structured interview	10(100%) recommend surgery
Wang et al. 2020 [12]	26	LeFort colpocleisis		4(15.4) VH 2(7.7) MUS	71.8±7.3	33	O: POP-Q ≤I S: PGI-I very much better/much better PFDI-20	O: 100% S: 100% PFDI-20 significantly improved
Park et al. 2019 [13]	95	LeFort colpocleisis			76.0	29	O: no descent beyond hymen S: satisfaction question	O: 94/95(98.9%) S: 75/78(96.2%) satisfied Body image improved
Cho et al. 2017 [14]	107	LeFort colpocleisis		9(8.4) MUS	75.5±5.8	7	O: POP-Q ≤II	O: 100(93.5%)
Dessie et al. 2017 [15]	54	Colpocleisis/colpectomy		1(1.9) VH 30(55.6) anti-UI procedure	79.9	10	O: POP-Q ≤I	O: 49(90.7%)
Wang et al. 2017 [16]	278	LeFort colpocleisis		231(83.1) VH 16(5.8) TLH 47(16.9) MUS	72.4±7.0	36	O: no recurrence S: satisfied PFDI-20, Body Image Scale (BIS)	O: 277(99.6%) S: 270(97%) 8(2.9%) dissatisfied (due to OAB, UI, vaginal bleeding after surgery) PFDI-20, BIS significantly improved
Crisp et al. 2016 [17]	61			No incontinence procedures	78.8±6	6	S: satisfied PFIQ-7, PFDI-20 Body Image Scale (BIS) Satisfaction with Decision Scale (SDS) VAS satisfaction	S: 55(90.2%) PFIQ-7, PFDI-20, BIS significally improved SDS 4.6±0.6 VAS 8.7±1.8
Katsara et al. 2016 [18]	20			6(30) VH	82.7	41	O: no recurrence	O: 100%
							Structured questionnaire on: QoL, body image, recommendation	15(75%) positive impact on QoL 2(10%) body image changed 90% recommend surgery
Ng and Chen 2016 [19]	22	LeFort colpocleisis		1(4.5) MUS 5(22.7) Kelly plication	81±5.5	48	S: satisfaction question	S: 15/16(93.8%)
Song et al. 2016 [8]	35	LeFort colpocleisis		6(17.1) VH 4(11.4) MUS	75.4±4.7	60	O: no recurrence S: PGI-Change satisfaction PFDI-20	O: 100% S: 33(94.3%) PFDI-20 significantly improved
Krissi et al. 2015 [43]	47	LeFort colpocleisis		7(14.9) MUS	77.3±8.2	15	O: POP-Q ≤1 S: according to symptoms	O: 38(80.9%) S: 43(91.5%)
Takase-Sanchez et al. 2015 [21]	77	70(91) complete vaginectomy 7(9) LeFort		19(24.7) VH 27(35.1) MUS 39(50.6) Kelly plication	79.3±7.0	30	O: no recurrence Satisfaction with Decision Scale (6-point scale)	O: 100% SDS 5.19±0.80
Vij et al. 2014 [22]	23	LeFort colpocleisis			78.7	36	O: no recurrence S: recommendation question P-QOL, ICIQ-UI SF, CRADI, POPDI	O: 21(91.3%) S: 21(91.3%) recommend surgery 1(4.3%) would no t recommend Questionnaires: low impact of the condition on QoL
Crisp et al. 2013 [23]	87	37(45.1) LeFort colpocleisis 45(54.2) Colpocleisis/colpectomy		9(10.8) VH 35(41.7) MUS 1(2.8) Kelly plication	79±5.8	1.5	PFIQ-7, PFDI-20, BIS SDS VAS satisfaction	PFIQ-7, PFDI-20, BIS significantly improved SDS 4.7±0.6 VAS 9.2±1.6
Eisenberg et al. 2013 [24]	16	Le Fort colpocleisis 9(56.2) prior H or at the time of colpocleisis		1(6.2) MUS	75.7	6.5	O: POP-Q ≤1	O: 10(62.5%)
Reisenauer et al. 2013 [25]	38	LeFort colpocleisis modified		21(55) VH	81.9±6.4	14	O: no prolapse to the hymen	O: 100%
							S: satisfaction question	S: 34(89.5%) 100% recommend surgery
Zebede et al. 2013 [26]	310	LeFort colpocleisis		2(1) VH 230(74.2) MUS 8(2.6) Kelly plication 6(1.9) bulking agent	81.3±5.3	10	O: POP-Q ≤1	O: 304(98.1%)
							S: descriptive scale of satisfaction	S: 288(92.9%)
Koski et al. 2012 [27]	53	39(73.6) total colpocleisis 14(26.4) Le Fort		2(3.8) VH 32(60.4) MUS	81	Office 9 Survey 31	O: no recurrence	O: 100%
							S: satisfaction question	S: 19/22(86.4%)
							S’: PGI-I very much better/better	S’: 20/22(90.9%)
							body image, body perception, UDI-6, POPDI-6	Body image worse 1(4.5%)
								POPDI-6 low POP bother
Yeniel et al. 2012 [28]	10	LeFort colpocleisis		1(10) MUS	74.9±4.5	6	O: no recurrence	O: 100%
							PQOL	PQOL significantly improved
Smith et al. 2011 [5]	210	LeFort colpocleisis 56(26.7) voiding dysfunction preoperatively		161(77) MUS	82.2±4.9	5	No symptoms of SUI in patients with MUS No voiding dysfunction	92.5% of MUS patients - continent 51/56(91%) resolution of preoperative voiding dysfunction
Abbasy et al. 2009 [6]	38			38(100) MUS	Median 79 (65–90)	3	O: POP-Q assessment Urinary retention: >100ml UDI-6, POPDI	O: 37/38(97.4%) 2(5.3%) urinary retention UDI-6, POPDI significantly improved
Fitzgerald et al. 2008 [29]	152	88(58) partial colpocleisis 64(42) total colpocleisis		12(8) H 55(36.2) MUS 2(1.3) xenograft suburethral sling 13(8.5) Kelly plication 1(0.7) periurethral collagen	79.3±5.6	12	O: POP-Q assessment Body Image and Satisfaction Questionnaire Short Form-36 (SF-36), PFDI, PFIQ	O: 75/103(73%) POP-Q ≤1 96/103(93%) POP-Q ≤2 S: 125(95%) very satisfied/satisfied PFDI, PFIQ significantly improved
Murphy et al. 2008 [30]	45	28(62.2) Le Fort colpocleisis 17(37.8) total colpocleisis		32(71.1) MUS	80.0±6.2	Office 6 Survey 17	O: no prolapse beyond hymen IIQ-7, UDI-6, Surgical Satisfaction Questionnaire (SSQ)	O: 42(93.3%) IIQ-7, UDI-6 significantly improved SSQ comparable between reconstructive and obliterative groups.
Agarwala et al. 2007 [2]	39			39(100) MUS	Median 82 (76–94)	24	S: satisfaction question SUI improvement question	S: 37/39(95%) quite satisfied 35/39(89.7%) improved of SUI
Barber et al. 2007 [31]	30	20(67) total colpocleisis 5(17) LeFort 5(17) partial colpocleisis		8(27) VH 13(43) MUS	77.8±5	12	O: no prolapse beyond hymen S: PGI-I much better/very much better PFDI-46, PFIQ, SF-36, Beck Depression Inventory (BDI)	O: 100% S: 14/30(47%) 11/30(37%) somewhat better PFDI, PFIQ, SF-36 significantly improved BDI no significantly change after surgery 90% recommend surgery
Hullfish et al. 2007 [32]	40			1(2.5) VH 14(35) anti-UI procedure	75.4±6.8	34	Satisfaction, and recommendation question UDI, IIQ	S: 38(95%) satisfied/very satisfied 39(97.5%) recommend surgery UDI, IIQ significantly improved
Deval 2005 [33]	30	Colpocleisis, hystero-colpectomy, suburethral plication, anterior, posterior colporrhaphy and high levator plication		30(100) VH 30(100) Kelly plication	78.1 ± 5.8	Office 8 Phone 35	O: no prolapse to the hymen S: satisfied/very satisfied	O: 30(100%) S: 22/25(88%) 25/25(100%) QoL improved
Glavind et al. 2005 [34]	42	25(59.5) LeFort colpocleisis 17(40.5) colpectomy			79	Office 3 Phone 46	O: no prolapse beyond hymen S: satisfaction question	O: 42(100%) S: 26/29(89.7%) satisfied
Wheeler et al. 2005 [35]	32	LeFort colpocleisis		5(15.6) MUS 13(40.6) Kelly plication 1(3.1) modified Pereya suspension	81.4±5.1	27.5	O: no prolapse to the hymen S: satisfaction question IIQ-7, UDI-6	O: 28/32(87.5%) S: 16/28(57%) completely satisfied, 8/28(29%) somewhat satisfied IIQ-7, UDI-6 significantly improved
FitzGerald and Brubaker 2003 [36]	64	63(98.4) LeFort colpocleisis 1(1.6) colpectomy		21(32.8) autologous fascia suburethral sling 12(18.8) Kelly plication	Median 78 (68–90)	3	O: no symptoms of POP no symptoms of SUI	O: 58/60(97%) 18/21(86%) SUI continent 3/21(14%) SUI persisted 3(14%) urinary retention, sling take down
Moore et al. 2003 [1]	30	Colpocleisis		30(100) MUS	79.2	19	O: no prolapse beyond introitus	O: 27/30(90%) 28(94%) SUI continent 2(6.6%) mild SUI
von Pechmann et al. 2003 [37]	92	Total colpocleisis		37(40.2) VH	-	Office 12 Phone 24	O: no prolapse to hymen S: satisfaction question	O: 90/92(97.8%) S: 56/62(90.3%) very satisfied/satisfied

Data presented as mean ± standard deviation, (range), or as stated elsewhere

*BDI* Beck Depression Inventory, *BIS* Body Image Scale, *CRADI* Colorectal Distress Inventory, *H* hysterectomy, *ICIQ-UI SF* International Consultation on Incontinence Questionnaire-Urinary Incontinence Short Form, *IIQ* Incontinence Impact Questionnaire, *MUS* midurethral sling, *O* objective, *OAB* overactive bladder, *PFDI* Pelvic Floor Distress Inventory, *PFIQ* Pelvic Floor Impact Questionnaire, *PGI-I* Patient Global Impression of Improvement, *POP* pelvic organ prolapse, *POPDI* Pelvic Organ Prolapse Distress Inventory, *POP-Q* Pelvic Organ Prolapse Quantification, *QoL* *P-QOL* perceived quality of life, *QoL* quality of life, *S* subjective, *SDS* Satisfaction with Decision Scale, *SF* short form, *SSQ* Surgery Satisfaction Questionnaire, *SUI* stress urinary incontinence, *TLH* total laparoscopic hysterectomy, *UDI* Urogenital Distress Inventory, *UI* urinary incontinence, *VAS* visual analog scale, *VH* vaginal hysterectomy

### Risk of bias

The data obtained are reported as a narrative review. We evaluated the methodological quality of the studies included using ROBINS-I: a tool for assessing the risk of bias in nonrandomized studies of interventions (version for cohort-type studies; Appendix 1) [38].

## Results

A total of 237 publications were identified. After removing the duplications, 173 records were screened. Full-text articles (*n* = 49) were assessed for eligibility (Fig. 1).

### Patient characteristics

Mean patient age ranged from 69.0 ± 8.0 [39] to 84 ± 3.1 years [40]. The oldest patients undergoing surgery were 95.9 [25] and 101 years [34]. In the studies by Krissi et al. [20] and Mueller et al. [41], women aged >80 years constituted 48.9% and 43% of the total patient population respectively. Colpocleisis may also be performed in younger women. In a large database of 4,776 subjects, colpocleisis was found to have been performed in 47 (0.9%) patients aged 20–39 years. The patient and the surgeon might choose the vaginal closure procedure over a reconstructive surgery to manage advanced POP in young women with serious comorbidities [41]. Between 90.2% and 100% of the patients undergoing colpocleisis were diagnosed with POP-Q stage ≥3. Subjects with POP-Q stage 2 constituted an insignificant percentage of the group, from 1.1% to 9.8% [6, 12, 23, 25, 26, 36, 37, 39]. A few studies either used other POP scales or did not clearly state which tools were used [32, 40, 42].

### Follow-up time

The longest follow-up (median: 5 years) was reported by Song et al., who used telephone contact, with “no recurrence” as the accepted criterion of anatomical success [8]. Studies that present the anatomical criterion using the POP-Q base it on the office visit. The longest follow-up study that included anatomical assessment is that by Wang et al. (33.1 ± 18.4 months [12]), but other authors had follow-ups of 16.9 ± 22.1 [10] and 14.8 ± 10.3 months [43]. Studies with the shortest follow-up (30 days) focus on perioperative complications [44, 45], the effect of frailty on postoperative complications [46], in addition to the morbidity and mortality associated with colpocleisis [41].

### Objective success

Anatomical success defined as POP-Q stage ≤1 ranged from 62.5% to 100% [7, 12, 15, 24, 26, 29, 43]. Eisenberg et al. reported an anatomical success rate of 62.5% (POP-Q ≤1), but the success rate increased to 100% when they extended the definition to POP-Q ≤2 [24]. A similar increase was found in a study by Fitzgerald et al.: 73% POP-Q ≤1 and 93% POP-Q ≤2 [29]. In accordance with the definition used by some authors—no prolapse beyond the hymen—the rates of anatomical success were 87.5% [35], 90% [1], 93.3% [30], 97.8% [37], 98.9% [13], and 100% [25, 31, 33, 34]. In turn, in studies that defined the successful outcome of colpocleisis as “no recurrence,” the rates were 91.3% [22], 99.6% [16], and 100% [8, 18, 21, 27, 28]. No symptoms of POP were reported by FitzGerald and Brubaker in 97% of the patients assessed [36].

Summarizing, if the recurrence rate is based on the POP-Q scale, patients with POP-Q stage ≥2 constitute even up to 27.2% of the study population after a mean follow-up of 12 months [29], or 37.5% after 6.5 months [24]. If the authors defined recurrence as POP-Q stage >2, they noted a recurrence rate of 6.5% after mean follow-up of 7.2 months [14]. In a study that analyzed factors related to recurrence, patients with recurrence had a longer duration of prolapse than those with a successful outcome of colpocleisis (24.6 ± 22.8 vs 8.0 ± 12.9 years, *p* = 0.02) [14]. Additionally, genital hiatus and total vaginal length were significantly associated with an increased risk of prolapse recurrence [43].

### Subjective success

Various studies employed different methods of subjective satisfaction assessment. On the PGI-I scale patients indicating “very much better” or “much better” are usually defined as “success.” The percentages of success in the analyzed studies ranged from 89.7% (26 out of 29) [10], 90.9% (20 out of 22) [27], to 100% (26 out of 26) [12]. In a study by Barber et al., only 47% of the women (14 out of 30) stated they were either “very much better” or “much better” on the PGI-I, whereas the additional 37% (11 out of 30) were “somewhat better.” At the same time, 90% of the subjects who underwent colpocleisis surgery stated that they would choose the same treatment again [31]. The PGI of Change revealed that 94.3% were satisfied subjects (33 out of 35) [8].

Analysis of PFDI comparing pre- and postoperative results indicated significant improvement in many studies on all subscales of the PFDI-20 [8, 10, 12, 16, 17, 23] or the PFDI-46 [31]. Although PFDI and PFIQ are complementary questionnaires, only a few authors used them simultaneously [17, 23, 29, 31]. The rates of success measured via the application of the general subjective satisfaction question ranged from 88% to 97.1% [12, 33]. In large studies, among >100 women, the subjective success rates were 92.9% (288 out of 310) [26], 97.1% (270 out of 278) [16], and 95% (125 out of 132) [29]. The lowest satisfaction rate was observed by Katsara et al. in 20 patients, of whom 75% reported a positive impact on their quality of life (QoL) [18]. Moreover, Wheeler et al. reported that 57% were “completely satisfied” (16 out of 28) and an additional 29% were “somewhat satisfied” patients (8 out of 28) [35]. From 90% [18, 31] to 100% [25] of the patients would undergo the same procedure again and 91.3% would recommend the surgery to others [22].

### Body image

The Body Image Scale (BIS) was applied by authors from China and the USA. In the former, mean (0.088 ± 0.155 vs 0.056 ± 0.101) and total (0.708 ± 1.239 vs 0.446 ± 0.812) BIS scores improved significantly during the long-term follow-up of a median of 3 years (*p* < 0.001) [16]. In the latter study, median BIS (*interquartile range*, IQR) score changed in two studies: 0.12 (0–0.6) vs 0 (0–0.2), *p* < 0.001 [17] and 0.25 (0–1.03) vs 0 (0–0.25), *p* < 0.001 [23]. Other methods of evaluation include questions or a pre-designed structured questionnaire to assess body image or body perception. Most patients (96%) were satisfied with the improvement in their body image [13], 82% reported that their body “felt better” [27]. In a study by Koski et al., after a mean follow-up of 31 months, 50% of all the patients reported “improved” body image, and 36% reported “no change” [27]. In a study by Fitzgerald et al., patient-reported body self-image 1 year after surgery compared with baseline was “improved” in 61% (80 out of 131), “the same” in 37% (49 out of 131), and “worse” in 2% of the patients (2 out of 131) [29].

On the other hand, unchanged body self-image, not altered by the procedure, was reported by 90% of women in the study by Katsara et al. and all patients in the study by Deval [18, 33].

### Regret rate

The loss of the ability to have penetrative vaginal intercourse after surgery remains an important aspect of colpocleisis. The issue is represented in the literature as “regret following colpocleisis.” In this study, regret has been subcategorized into “general decision regret” and “regret of coital ability.” The general regret rate ranged from 0% [1, 8, 16, 25, 34] to 13.8% [23]. The main reasons included POP recurrence, urinary incontinence, or postoperative complications. Only a few authors have addressed regret over the loss of sexual function. No regret regarding coital ability was reported by many studies [1, 7, 11, 18, 22, 25, 35, 43], but coital regret was reported too: from 1.15% to 12.9% [17, 23, 32, 37]. In a study by Fitzgerald et al., 3% (2 out of 79), 87% (69 out of 79), and 10% of the women (8 out of 79) reported “worse,” “the same,” or “better” sexual function respectively, after 1 year of follow-up [29]. Deval found that 52% of the women (13 out of 30) remained sexually active after colpocleisis by means of clitoral stimulation (Table 2) [33].

**Table 2:** Studies presenting regret rate following colpocleisis

References	*N*	General decision, regret, reason	Regret regarding coital ability
Kato et al. [7]	20	No data	0%
Wadsworth and Lovatsis [11]	10	1 (10%) – UUI de novo	0%
Park et al. [13]	95	3 (3.8%) – postoperative complications (rectal prolapse, recurrence, feeling of a bearing down sensation)	No data
Wang et al. [16]	278	0%	No data
Crisp et al. [17]	61	6 (9.8%)	1 (1.6%)
		DRS^a^ 1.5 ± 0.7	
Katsara et al. [18]	20	2 (10%) – urinary problems	0%
Song et al. [8]	35	0%	No data
Krissi et al. [20]	47	No data	0%
Takase-Sanchez et al. [21]	77	3 (3.9%)	No data
		DRS^b^ 1.75 ± 0.9	
Vij et al. [22]	23	1 (4.3%) – recurrence	0%
Crisp et al. [23]	87	12 (13.8%)	1 (1.15%)
		DRS^a^ 1.32 ± 0.6	
Reisenauer et al. [25]	38	0%	0%
Fitzgerald et al. [29]	132	No data	2/80 (3%) worse sexual function
Hullfish et al. [32]	40	4 (10%)	2 (5%)
Deval [33]	30	No data	0%
			13 (52%) remained sexually active by clitoral stimulation
Glavind and Kempf [34]	42	0%	No data
Wheeler et al. [35]	32	3 (9.3%) – 2 recurrence, 1 SUI	0%
Moore and Miklos [1]	30	0%	0%
von Pechmann et al. [37]	62	No data	8/62 (12.9%)

*DRS* Decision Regret Scale, *SUI* stress urinary incontinence, *UUI* urgency urinary incontinence

^a^Five-point scale

^b^Six-point scale

The DRS showed a mean score of 1.32 ± 0.59 [23] and 1.52 ± 0.69 [17], at 6 and 24 weeks respectively, signifying very little regret. A six-item modified DRS was used by Takase-Sanchez et al., resulting in a score of 1.75 ± 0.90 after 2.5 years of follow-up [21].

### Goal accomplishment

The level of satisfaction with surgery depends on the achievement of the goals set before the procedure. Most women “agreed” or “strongly agreed” that their pre-surgery goals were met for vaginal pressure (100%), urinary incontinence (84.9%), bladder emptying (76.4%), urinary frequency/urgency (91.2%), physical activity (88.6%), restoration of normal anatomy (95%), colorectal symptoms (65.0%), and self-image (96.9%) [32]. Goal achievement correlated with the postoperative Urogenital Distress Inventory (UDI) (*r* = −0.45, *p* = 0.003), although not the Incontinence Impact Questionnaire (IIQ) [32]. Patient goals and preferences may be more important than standardized objective outcome measures, especially in terms of regret and satisfaction. Linear regression models have identified preoperative sexual activity as the only independent predictor of more decision regret after obliterative surgery (β coefficient 1.68, *p* < 0.001), reoperation for any reason as an independent predictor of lower satisfaction (β, −0.24; *p* = 0.04), and the patient-reported reason for elective obliterative surgery of “not interested in pessary” as a predictor of higher satisfaction (β, 0.30, *p* = 0.01) [21].

### Effects of colpocleisis on bowel symptoms

Among women undergoing colpocleisis, at least one bothersome bowel symptom was present in 77% of the subjects preoperatively, including obstructive symptoms (17–26%), incontinence (12–35%), and pain/irritation (3–34%) [47]. Colorectal Distress Inventory (CRADI) scores decreased significantly after colpocleisis, resulting in lower bother from colorectal symptoms [8, 10, 12, 16, 29]. At 1 year of follow-up, the symptoms were less prevalent, and the scores for the colorectal domains of the QoL questionnaires (CRADI and Colo-Rectal-Anal Impact Questionnaire) significantly improved. Low rates of de novo symptoms (0–14%) were reported [47]. The change in CRADI scores did not correlate with decision regret regarding obliterative surgery [21]. Therefore, colorectal symptoms after colpocleisis were not responsible for the feelings of regret after surgery.

### Types of colpocleisis, concomitant hysterectomy

The LeFort colpocleisis is the most commonly performed surgery. It is usually conducted in women with a preserved uterus, with no concomitant hysterectomy, but it has also been performed on the vaginal vault [5, 8, 12–14, 16, 19, 22, 24, 28, 43]. In many studies, colpocleisis is combined with hysterectomy.

In a study comparing total colpocleisis with vaginal hysterectomy (VH) versus total colpocleisis of the vaginal vault, VH was associated with a significant increase in absolute change in hematocrit (11.9% vs 9.5%, *p* = 0.01) and the need for transfusion (35.1 vs 12.7%, *p* = 0.02) [37], which was confirmed in a later study, in 2019. Women undergoing total colpocleisis with VH had a higher hemoglobin drop (15 ± 12 vs 11 ± 9 g/L, *p* = 0.006) and red blood cell (RBC) loss (196 ± 150 vs 140 ± 117, *p* = 0.01) than women with colpocleisis alone [48]. Hill et al. reported higher blood loss (253 vs 146 ml, *p* = 0.01), higher transfusion rate (9.3% vs 4.3%, *p* = 0.02), and longer operative time (144 vs 111 min, *p* < 0.001) with concomitant VH [42]. In the study by Fitzgerald et al., the difference in favor of the partial colpocleisis as compared with the total colpocleisis remained visible even after excluding patients who underwent concurrent hysterectomy. The estimated blood loss in the total colpocleisis group was significantly greater than in the partial colpocleisis group (149 ± 127 ml vs 90 ± 56 ml, *p* = 0.002) [29]. In an American study with 1,027 patients, VH at the time of colpocleisis was the only variable independently associated with serious medical complications (*p* < 0.05) [44].

**Fig. 2:**
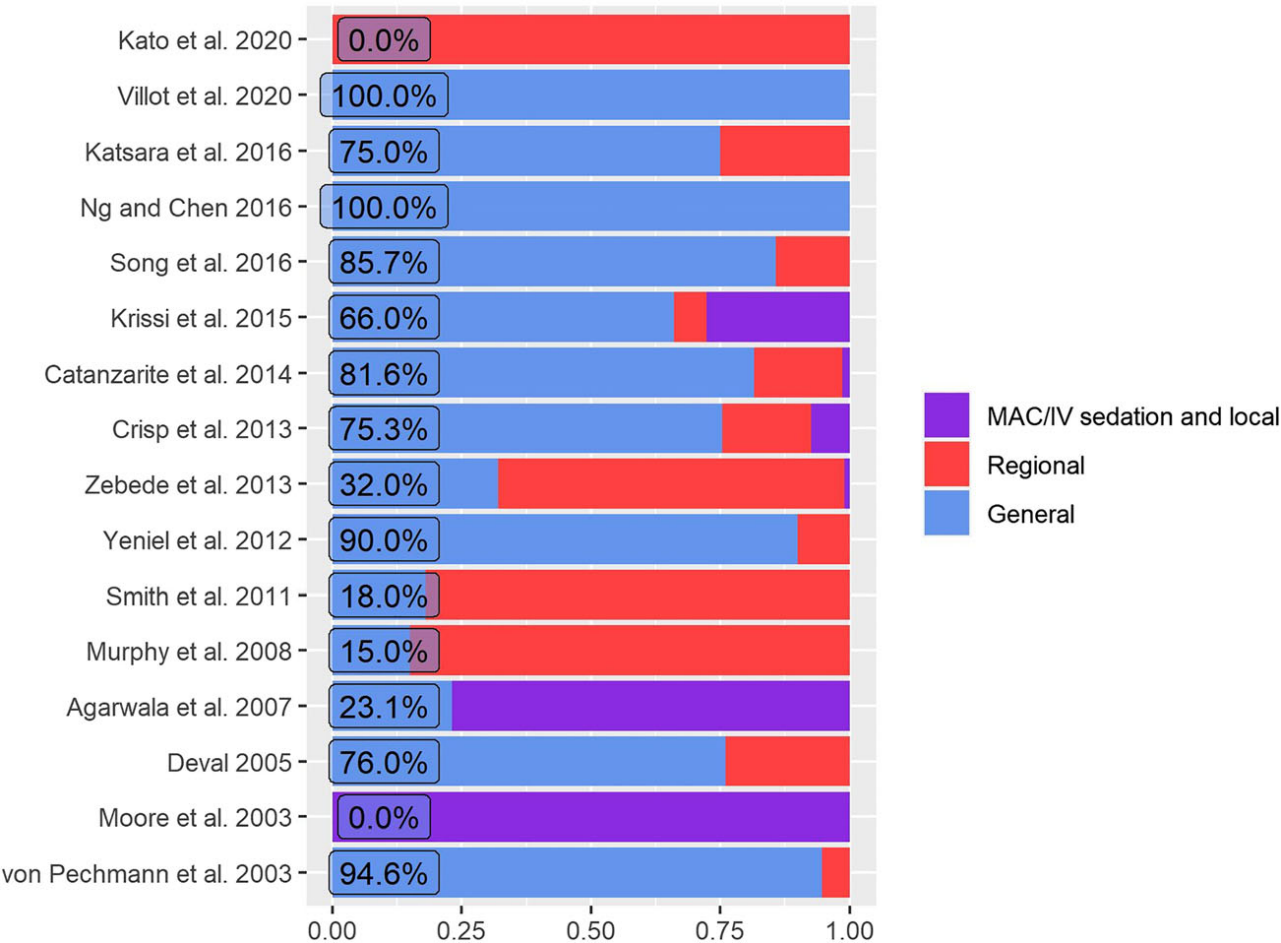
Types of anesthesia used in the studies analyzed. *MAC/IV* monitored anesthesia care or intravenous sedation. Percentage values presented for general anesthesia

Types of anesthesia also vary between studies. In recent studies, monitored anesthesia care and intravenous sedation have been used only on rare occasions. General anesthesia remains the most common type of anesthesia applied (Fig. 2).

### Colpocleisis and midurethral slings

Smith et al. performed MUS concomitantly in 161 patients, and found no symptoms of SUI in 92.5% of the patients [5]. According to other authors, 86.8% (33 out of 38) [6], 89.7% (35 out of 39) [2], and 94% (28/30) [1] of the patients were continent after MUS with colpocleisis. Agarwala et al., apart from MUS (28 polypropylene mesh and 11 xenografts), inserted the remaining mesh strips from the sling between the pubocervical and rectovaginal fascia, providing extra support [2]. An autologous fascia suburethral sling was used by Fitzgerald et al., who reported that 86% of the patients were continent (18 out of 21) and that 14% of the women (3 out of 21) had persistent SUI [36].

Implantation of MUS in patients with severe prolapse requires thorough analysis of pre- and postoperative voiding dysfunction. Post-void residual (PVR) of >100 ml preoperatively was reported for 31.6% (12 out of 38) [6] and 45% (95 out of 210) [5] of all women undergoing colpocleisis. Preoperative voiding dysfunction, defined as PVR of >100 ml and the presence of abnormal voiding or abnormal urinary stream, was diagnosed in 26.7% (56 out of 210) [5] and 35.8% (111 out of 310) [26] of the patients. In a study by Smith et al., MUS implantation resulted in de novo voiding dysfunction, which persisted for over 6 weeks after surgery, in 1.9% of the patients (3 out of 161), with a 0.6% sling revision rate [5]. Other authors reported 0% [2, 45], 3% (1 out of 30) [1], 3.7% (1 out of 27) [21], and 14% (3 out of 21) [36] of suburethral release/revision of a sling due to urinary retention.

Catanzarite et al. conducted an analysis of the 30-day complication rates after colpocleisis, dichotomizing the subgroups based on MUS insertion. In the two groups of 92 and 191 women with and without concomitant sling placement, they observed similar rates of complications (7.9% vs 8.7%, *p* = 0.81), urinary tract infection (UTI; 5.8% vs 7.6%, *p* = 0.55), return to the operating room (2.1% vs 2.2%, *p* = 0.97), and mortality (0% vs 1.1%, *p* = 0.15) [45].

Significant improvement in QoL questionnaires assessing impact and distress caused by the lower urinary tract symptoms was reported after colpocleisis with MUS [6, 30] or other anti-incontinence procedures [32, 35].

### Obliterative versus reconstructive surgery

Several studies compared obliterative and reconstructive surgery. The patients undergoing obliterative surgery were significantly older, with more advanced POP, and higher odds of being frail [39, 46]. Data on the operative parameters and complications remain inconclusive. Some authors found no differences [39], whereas others report greater severity of complications in women undergoing reconstructive surgery [15]. A study from 2016 confirms a higher rate of grade III Clavien–Dindo scale complications in the reconstructive surgery group (16.9% vs 13.0%, *p* = 0.02) [15]. Sung et al. also reports a higher risk of complications (24.7% vs 17.0%, *p* < 0.01) [49]. In a study with 12,731 women undergoing POP repair, the type of the procedure was not associated with higher or lower odds of postoperative complications [46]. However, the patients undergoing obliterative surgery were at a higher risk (OR 22; 95% CI, 2.3–196; *p* < 0.002) for full-thickness rectal prolapse [50].

Beyond the aforementioned, mean duration of obliterative surgery was shorter than for reconstructive surgery (150 ± 23 vs 180 ± 16 min, and 1.92 vs 2.66 h, *p* < 0.001) [31, 46]. Furthermore, the obliterative surgery group had a significantly shorter hospital stay in a study by Petcharopas et al., whereas no difference was reported by Barber et al. [31, 39]. A higher number of other than general anesthesia or monitored anesthesia care (14.5% vs. 3.4%) was also noted [46]. The patients who underwent colpocleisis received the lowest mean morphine milligram equivalent (MME) (137.6 ± 99.8 mg) than other reconstructive surgeries, with the highest mean MME prescribed to patients undergoing laparoscopic uterosacral ligament suspension (214.1 ± 87.5 mg) (*p* < 0.0001) [51]. In addition, hemoglobin drop in colpocleisis was significantly lower than in the reconstructive procedures (*p* < 0.05) [48].

However, most studies have unanimously confirmed that improvement in condition-specific QoL and postoperative patient satisfaction measures were comparable in women with prolapse undergoing either reconstructive or obliterative surgery [30, 31, 39].

### Complications

The rates of complications and Intensive Care Unit (ICU) admission were low, with mean rates of 6.8% and 2.8% respectively [41]. Return to the operating room was reported and ranged between 0% and 8.1% [10].

Urinary tract infection is the most common postoperative complication [44, 45]. During the first 30 peri-operative days, according to the definition applied in the American College of Surgeons National Surgical Quality Improvement Program (ACS NSQIP) surgical risk calculator, the rate of UTI was 4.3% in a study among 1,027 subjects [44], and 5.8% vs 7.6% (*p* = 0.55) in a study on colpocleisis without versus with a concomitant sling placement [45]. In turn, Hill et al., who defined UTI as symptoms of urgency or frequency or dysuria and/or a positive urine culture, reported 34.7% within the first 6 weeks after the surgical procedure. They did not confirm differences in the prevalence of UTI depending on the type of surgery: colpectomy, VH plus colpectomy, or LeFort colpocleisis (*p* = 0.45) [42]. Definitions of UTI vary across the literature, and not all authors include positive urine culture as crucial for the diagnosis. Indeed, sometimes symptoms associated with the initiation of antibiotics are believed to be sufficient to make the diagnosis. In a study with 310 patients, the rate of UTI confirmed with urinary culture was 9% [26]. Initiation of antibiotics for suspected UTI with no culture resulted in the rate of 26.1% [22]. Sifuentes et al., who based their estimates on both these definitions, detected a UTI rate of 25.4% [40]. Fitzgerald et al. reported urogenital symptoms during 3 months of follow-up in as many as 45% of the patients, stating that these were mostly UTIs [29].

Rectal or bowel injuries are rare complications, absent in most studies or found in single cases [5, 10, 18], or in a few patients. These constituted 0.8% [42] and 0.6% [26] of the group (Table 3). Rectal injuries are usually sutured during surgery [10, 18]. Zebede et al. reported bowel thermal injury during additional procedures, or unrecognized large bowel injury occurring at the time of suprapubic catheter placement [26]. Intraoperative injury to the urinary tract, if it occurs, most often includes bladder injuries reported in 0.7% [29], 1.9% [27], and 9.1% of the patients [18]. Zebede et al. observed 2 bladder perforations (0.6%) secondary to the placement of needles for sling procedures [26]. Apart from that, Hill et al. reported urethral and ureteric injury in the group of colpectomy with vaginal hysterectomy [42], whereas Fitzgerald et al. mentioned ureteric kinking and urethral injury [29]. Von Pechmann et al. administered intravenous indigo carmine during total colpocleisis in order to cystoscopically confirm ureteral patency, and found a rate of 4.3% of the total group of reversible ureteral occlusion: 1 patient in the nonhysterectomy group (1.8%) and 3 patients in the hysterectomy group (8.1%) (*p* = 0.3) [37].

**Table 3:** Intra- and postoperative complications after colpocleisis

References	*n*	Urinary tract infection	Bladder/ureter/ urethra injury	Rectal /bowel injury	Prolapse recurrence, if stated reoperation. Rectal prolapse	Other complications
Kato et al. [7]	54	1 (1.9%)	None	None	4 (7.4%) reoperation	1 (1.9%) return to OR (bleeding), 2 (3.7%) peritoneal opening
Villot et al. [10]	37	ND	None	1 (2.7%)	None	3 (8.1%) return to OR (C–D 3b), 1 (2.7%) pararectal abscess (C–D 3a), 16 (43.2%) C–D 2
					1 (2.7%) rectal prolapse	
Wang et al. [12]	26	1 (3.8%)	None	None	None	4 (15.4%) C–D 2
Cheng et al. [48]	176	ND	ND	ND	ND	1 (0.6%) hematoma
Park et al. [13]	95	1 (1.1%)	None	None	1 (1.1%)	2 (2.1%) blood transfusion, 1 (1.1%) perineal wound infection
					1 (1.1%) rectal prolapse	
Sifuentes et al. [40]	126	32 (25.4%)	None	None	ND	4 (3.2%) serious (pulmonary embolism, sepsis, DVT, reintubated)
Petcharopas et al. [39]	93	7 (7.53)	None	None	ND	1 (1.1%) return to OR, 2 (2.2%) pelvic abscess, 14 (15.1%) pyrexia
Bochenska et al. [44]	1027	44 (4.3%)	None	None	ND	12 (1.2%) serious (4 sepsis, 2 CVA, 2 SSI, 2 MI, 1 DVT, 1 cardiac arrest)
Cho et al. [14]	107	6 (5.6%)	None	None	7 (6.5%)	5 (4.7%) postoperative bleeding, 3 (2.8%) blood transfusion, 2 (1.9%) vulvar edema
					2 (1.9%) fecal incontinence	
Dessie et al. [15]	54	3 (5.6)	None	None	5 (9.3%) recurrence & reoperation	3 (5.6%) vaginal bleeding
					3 (5.6) fecal incontinence	
Wang et al. [16]	278	12 (4.3%)	None	None	1 (0.4%) reoperation	8 (2.9%) vaginal vault hematoma, 2 (0.7%) atrial fibrillation
Crisp et al. [17]	61	ND	ND	ND	1 (1.6%)	ND
Hill et al. [42]	245	85 (34.7%)	4 (1.6%)	2 (0.8%)	ND	3 (1.2%) VTE, 12 (4.9%) blood transfusion, 1 (0.4%) mortality, 9 (3.7%) bleeding, 6 (2.5%) hematoma, 2 (0.8%) abscess, 10 (4.1%) pulmonary events, 5 (2.0%) cardiac events, 4 (1.6%) ileus
Katsara et al. [18]	44	None	4 (9.1%)	1 (2.3%)	None	2 (5.4%) blood transfusion
Ng and Chen [19]	22	None	None	None	None	None
Song et al. [8]	35	1 (2.9%)	None	None	None	1 (2.9%) hematoma, 1 (2.9%) SSI
Krissi et al. [20]	47	2 (4.2%)	None	None	9 (19.1%)	None
Mueller et al. [41]	4,776	ND	ND	ND	ND	325 (6.82%) complication rates, 133 (2.8%) ICU admission, 7 (0.15%) mortality
Takase-Sanchez et al. [21]	77	ND	ND	ND	None	1 (3.7%) sling revision
Catanzarite et al. [45]	283	18 (6.4%)	None	None	ND	1 (0.4%) mortality, 6 (2.1%) return to OR, 2 (0.7%) SSI, 1 (0.4%) pneumonia, 1 (0.4%) stroke, 1 (0.4%) blood transfusion, 2 (0.8%) sepsis, 1 (0.4%) pulmonary embolism
Vij et al. [22]	23	6 (26.1%)	None	None	2 (8.7%)	1 (4.4%) SSI, 1 (4.4%) bleeding
Crisp et al. [23]	87	ND	None	None	ND	2 (2.7%) return to OR, 1 (1.4%) blood transfusion, 1 (1.4%) infection
Eisenberg et al. [24]	16	None	None	None	6 (37.5%) POP-Q ≥ 2	None
Reisenauer et al. [25]	38	8 (21%)	None	None	None	2 (5.3%) hematoma, 1 (2.6%) return to OR, 1 (2.6%) pyometra (hysterectomy after 6 months)
Zebede et al. [26]	310	25 (9%)	2 (0.6%)	2 (0.6%)	6 (1.9%)	1 (0.3%) uterine perforation during hysteroscopy, 4 (1.3%) mortality (1 MI, 2 pulmonary embolism, 1 sepsis), 1 (0.3%) hematoma, 2 (0.6%) cardiovascular event, 1 (0.3%) DVT
Koski et al. [27]	53	None	1 (1.9%)	None	None	1 (1.9%) blood transfusion (C–D 2), 1 (1.9%) pulmonary embolism (C–D 2), 1 (1.9%) clot evacuation (C-D 3b)
Yeniel et al. [28]	10	ND	ND	ND	None	None
Smith et al. [5]	210	ND	ND	1 (0.5%)	ND	1 (0.5%) superficial cellulitis, 1 (0.6%) sling revision, 1 (0.6%) removal of infected sling after 2 years
Abbasy et al. [6]	38	None	None	None	1 (2.6%)	2 (5.3%) readmissions (1 congestive heart failure, 1 vomiting)
Fitzgerald et al. [29]	152	68 (45%) in 3 months	3 (2%)	None	28/103 (27.2%) POP-Q ≥ 2	46/152 (30.3%) during initial hospitalization (1 pulmonary edema, 6 cardiovascular events, 2 SSI)
					1 (1.0%) rectal prolapse	
Murphy et al. [30]	45	ND	ND	ND	3 (6.7%)	1 (2.2%)
Agarwala et al. [2]	39	ND	None	None	2 (5.1%) reoperation	2 (5.1%) graft excisions (exposure)
Barber et al. [31]	30	None	None	None	None	1 (3%) blood transfusion, 1 (3%) return to OR, 1 (3%) pelvic abscess
Hullfish et al. [32]	40	4 (4.3%)	None	None	2 (2.1%)	1 (2.5) DVT, 1 (2.5%) postoperative bleeding, 1 (2.5%) atypical chest pain, 1 (2.5%) atrial fibrillation, 1 (2.5%) endometrial cancer in the specimen
					1 (2.5%) rectal prolapse	
Deval [33]	30	8 (26.6%)	None	None	None	1 (3.3%) cardiovascular, 1 (3.3%) pulmonary embolism
Glavind and Kempf [34]	42	ND	None	None	None	2 (4.8%) (1 return to OR with bleeding, 1 SSI)
Wheeler et al. [35]	32	ND	ND	ND	4 (7.4%)	ND
FitzGerald et al. [36]	64	ND	ND	ND	2/60 (3%)	1 (1.6%) death multisystem organ failure, 2 (3.1%) bleeding, 3/21 (14%) slings release
Moore and Miklos [1]	30	None	None	None	3 (10%) reoperation	1 (3%) MI, 1 (3%) sling release
von Pechmann et al. [37]	92	ND	4 (4.3%) ureteral occlusion	None	2 (2.2%)	20 (21.7%) blood transfusion, 2 (5.4%) converted to laparotomy (bleeding), 1 (1.1%) death after 28 days (metastatic lung cancer)
					2 (2.2%) rectal prolapse	

*C-D* Clavien–Dindo classification, *CVA* cerebrovascular accident, *DVT* deep vein thrombosis, *MI* myocardial infarction, *ND* no data, *OR* operating room, *SSI* surgical site infection, *VTE* venous thromboembolism

### Mortality

Based on the data from 145 US medical centers, the 30-day mortality rate was 0.15% [41]. In the single-center studies, the rates were 0.4% [42] and 1.1% due to pulmonary complications at 28 days postoperatively in one patient with the preoperative diagnosis of metastatic lung cancer [37], and 1.6% due to multisystem organ failure unrelated to surgery at 3 weeks after surgery [36]. Zebede et al. reported 4 deaths (1.3%), i.e., 2 pulmonary emboli, 1 sepsis and multiorgan failure after bowel injury, and 1 myocardial infarction that occurred 42 days after surgery [26]. In a multicenter study among 152 patients, the rate was 0.65%, with one death 5 months after surgery as a result of sepsis and congestive heart failure [29].

### Other risk factors

Mueller et al. conducted hospital volume analysis and determined the annual case volume as low <5, medium 5–10, and high with >11 cases per year. High-volume centers had lower ICU admission and complication rates as well as a shorter stay. The following variables were significant predictors of higher complication rates: lower age (*p* < 0.002), lower center volume (*p* < 0.02), and higher number of comorbidities (*p* < 0.0001). In that US multicenter study, stratification by provider specialty demonstrated higher complication rates among obstetrician/gynecologists and urologists than among urogynecologists [41]. In a retrospective analysis of calculator-computed risk, the patients with preoperative use of antiplatelets (clopidogrel or acetylsalicylic acid >81 mg) were nearly 5-fold more likely to experience complications than patients with no antithrombotic medication (adjusted OR 4.84; 95% CI, 1.72–13.60; *p* = 0.002). What is more, patients with hypertension were 4.25-fold more likely to experience a complication than those without hypertension (adjusted OR 4.24; 95% CI, 1.31–13.720; *p* = 0.016) [40].

## Discussion

This review of the literature allows the conclusion that colpocleisis is a safe procedure, with a high rate of anatomical and subjective success. Regret over surgery, if reported, is mostly associated with pelvic floor symptoms, especially urinary symptoms and/or the necessity for reoperation. A number of authors have confirmed the safety of the concomitant sling placement, although we lack randomized studies on the matter [2, 5, 6]. Bearing in mind that distress related to urinary incontinence is an independent predictor of lower success rate for colpocleisis, it seems advisable to perform concomitant anti-incontinence procedures [21]. Still, it is vital to keep track of the reported rates for postoperative voiding dysfunction. Reoperations due to recurrent POP remain relatively rare (up to 10%) [1], which is a comparable or even lower rate of relapses than in the case of other native tissue repairs. Many studies assess the distress caused by colorectal symptoms in women with POP. Most of the preoperative symptoms resolve after colpocleisis [47], although one study confirmed a higher risk of full-thickness rectal prolapse in patients who underwent obliterative surgery [50]. The debate concerns whether the increased proportion of rectal prolapse is due to a lack of a preoperative diagnosis or whether the colpocleisis itself increases intra-abdominal pressure and causes prolapse through the anorectal hiatus [50]. More extensive preoperative diagnostics of obstructed defecation syndrome could also be implemented [52].

It is essential to differentiate between the reasons for decision regret. Most studies have analyzed regret over coital ability. More detailed analyses found that a group of patients remained sexually active after colpocleisis by means of clitoral stimulation [33]. In turn, preoperative sexual activity was associated with greater decision regret regarding obliterative surgery [21]. As for the reconstructive surgery, regret regarding the decision for surgery resulted from symptomatic failure or the need for retreatment [53].

In this review, we make no comparison of total complication rates, which are based on diversified criteria that differ among studies. Serious intraoperative complications remain infrequent. Still, detailed data on comorbidities and mortality rates in that group of patients have been reported. An interesting study on frailty among patients undergoing pelvic floor surgery revealed that surgeons tend to select women for colpocleisis based on age, but it is frailty that has a stronger association with postoperative complications [46]. To support this, in the group of older women, lower rates of complications after colpocleisis were reported [45].

The rates of concomitant VH also differ across the centers, but the procedure is typically associated with a risk of higher blood loss [37, 48] and prolonged operative time [44]. In turn, according to different decision analysis models, the protective value of VH against unanticipated pathology and malignancy in the uterus can be observed only in younger women (<40 years of age) [3]. Therefore, indications for concomitant hysterectomy should be individually assessed. LeFort colpocleisis, which is frequently called “partial colpocleisis,” is performed not only in women with a preserved uterus but also on the vaginal cuff. It is not until a detailed description of the procedure is known that the exact type of surgery can be determined. This review of the literature revealed that the nomenclature used by various authors is inconsistent; thus, the current terminology report [4], which unifies the terminology of obliterative surgeries, is especially useful. It is essential to use uniform descriptions of the obliterative surgeries to allow comparison of results across studies.

When counseling patients on the choice of surgical treatment for POP, colpocleisis is a good option for some of them. However, it is necessary to carry out a detailed interview informing them of the obliterative nature of the method and its effect on sexual function. Prior to surgery, it is essential to carefully analyze other pelvic floor disorders, considering the possible consequences. Detailed analysis of occult SUI may result in establishing a future therapeutic plan. The information about colorectal symptoms after surgery may encourage patients to become aware of and report de novo symptoms.

The majority of the reports were retrospective, with only a small number of prospective studies [17, 23, 29, 31, 47]. The limitations of this review include small samples in some of the studies, different terminology and variables in surgical technique, relatively short follow-up, and diverse success assessment methods, very often performed via telephone survey. Most of the studies had a moderate risk of bias at the pre-intervention level. It should be noted that the patients discussed in these studies had undergone different concomitant procedures (usually anti-incontinence procedures), therefore confounding the effect of the intervention. Furthermore, success assessment induced a serious risk of bias in the post-intervention part, as most of the measurements of outcomes were patient-reported or performed by surgeons. Only a few studies had blinded assessors for success evaluation. Additionally, the selection and publication bias of this synthesis was not performed.

## Conclusions

Contrary to expectations, this review of the literature provides evidence that, rather than being a historical oddity, colpocleisis is very much present in the twenty-first-century pelvic floor surgery toolbox. The number of studies and the sample size in multicenter studies has revealed the actual percentage of patients undergoing this procedure all over the world. Despite having been introduced almost 150 years ago, colpocleisis continues to meet patient expectations regarding POP management owing to the low risk of intra- and postoperative complications.

## Supplementary Information

ESM 1 (DOCX 20 kb)
