# Supplementary material for: Colpocleisis as an obliterative surgery for pelvic organ prolapse: is it still a viable option in the twenty-first century? Narrative review
Source: Int Urogynecol J. 2021 Aug 18;33(1):31–46. doi: 10.1007/s00192-021-04907-7 (PMC8739283; doi:10.1007/s00192-021-04907-7)
Supplement: Supplementary file 1 — (DOCX 20 kb) [file 192_2021_4907_MOESM1_ESM.docx]

**Table A1** ROBINS-I: a tool for assessing risk of bias in nonrandomized studies of interventions

| **Authors/ Study** | Pre-intervention | | At intervention | Post-intervention | | | | ROBINS-I overall |
| --- | --- | --- | --- | --- | --- | --- | --- | --- |
|  | Confounding | Selection | Classification of interventions | Deviations from interventions | Missing data | Measurement of outcomes | Selection of the reported results | Risk of bias |
| Kato et al. 2020 | Moderate | Low | Low | Low | Low | Serious | Low | Serious |
| Villot et al. 2020 | Serious | Low | Low | Low | Moderate | Serious | Moderate | Serious |
| Wadsworth et al. 2020 | Low | Low | Low | Low | Low | Serious | Low | Serious |
| Wang et al. 2020 | Moderate | Low | Low | Low | Low | Serious | Low | Serious |
| Park et al. 2019 | Low | Low | Low | Low | Moderate | Serious | Low | Serious |
| Cho et al. 2017 | Moderate | Low | Low | Low | Low | No information | Low | Moderate |
| Dessie et al. 2017 | Moderate | Low | Low | Low | Low | No information | Low | Moderate |
| Wang et al. 2017 | Moderate | Moderate | Low | Moderate | Low | Serious | Low | Serious |
| Crisp et al. 2016 | Low | Moderate | Low | Low | Low | Serious | Low | Serious |
| Katsara et al. 2016 | Moderate | Moderate | Low | Low | Moderate | Serious | Low | Serious |
| Ng and Chen 2016 | Moderate | Low | Low | Low | Moderate | Serious | Low | Serious |
| Song et al. 2016 | Moderate | Low | Low | Low | Low | Serious | Low | Serious |
| Krissi et al. 2015 | Moderate | Low | Low | Low | Low | Serious | Low | Serious |
| Takase-Sanchez et al. 2015 | Low | Low | Low | Low | Low | Serious | Low | Serious |
| Vij et al. 2014 | Low | Moderate | Low | Low | Low | Serious | Low | Serious |
| Crisp et al. 2013 | Moderate | Low | Low | Low | Serious | Serious | Low | Serious |
| Eisenberg et al. 2013 | Low | Low | Low | Low | Low | Serious | Low | Serious |
| Reisenauer et al. 2013 | Moderate | Moderate | Low | Low | Low | Serious | Low | Serious |
| Zebede et al. 2013 | Moderate | Low | Low | Moderate | Low | Serious | Low | Serious |
| Koski et al. 2012 | Moderate | Low | Low | Low | Moderate | Serious | Low | Serious |
| Yeniel et al. 2012 | Moderate | Low | Low | Low | Low | Serious | Low | Serious |
| Smith et al. 2011 | Moderate | Low | Low | Low | Low | Serious | Low | Serious |
| Abbasy et al. 2009 | Moderate | Low | Moderate | Low | Low | Serious | Low | Serious |
| Fitzgerald et al. 2008 | Moderate | Low | Low | Low | Low | Serious | Low | Serious |
| Murphy et al. 2008 | Moderate | Low | Low | Low | Moderate | Serious | Low | Serious |
| Agarwala et al. 2007 | Moderate | Low | Low | Low | Low | Serious | Low | Serious |
| Barber et al. 2007 | Moderate | Low | Low | Low | Low | Serious | Low | Serious |
| Hullfish et al. 2007 | Moderate | Moderate | Low | Low | Low | Serious | Low | Serious |
| Deval 2005 | Moderate | Low | Low | Low | Moderate | Serious | Low | Serious |
| Glavind et al. 2005 | Low | Low | Low | Low | Low | Serious | Low | Serious |
| Wheeler et al. 2005 | Moderate | Moderate | Low | Low | Low | Serious | Low | Serious |
| FitzGerald and Brubaker 2003 | Moderate | Low | Low | Low | Low | No information | Low | Moderate |
| Moore et al. 2003 | Moderate | Low | Low | Low | Low | Serious | Low | Serious |
| von Pechmann et al. 2003 | Low | Low | Low | Low | Moderate | Serious | Low | Serious |
